# Patent quality and trade credit: Based on the perspective of knowledge breadth

**DOI:** 10.1371/journal.pone.0335515

**Published:** 2025-10-31

**Authors:** Yihan Li, Man Wang

**Affiliations:** School of Accounting, Management Accounting Research Center, Dongbei University of Finance and Economics, Dalian, China; Shandong University of Science and Technology, CHINA

## Abstract

Firms often rely on their own unique knowledge to obtain profits, but the reproducibility of knowledge will weaken economic interests, so firms adopt patents to establish exclusivity to clarify the ownership of profit rights. However, patents are only a form, and what kind of knowledge is contained behind them is the key to whether a firm can obtain and how much economic benefit it can obtain. In order to protect intellectual property rights in an all-round way, firms often hold a lots of patents, forming a patent matrix containing multiple cross-knowledge. The more complex the knowledge connotation of the patent matrix, the more difficult it is to be imitated, and the better the protection benefits of patents, forming high-quality patents. This study selects China’s A-share listed companies from 2006 to 2023 as the sample, utilizes patent acquisition data of listed firms, and measures corporate patent quality from the perspective of knowledge breadth—the wider the knowledge breadth embedded in patents, the higher the patent quality. Based on this framework, this study investigates how patent quality, measured by knowledge breadth, influences firms’ access to trade credit. The findings reveal that improvements in corporate patent quality significantly enhance access to trade credit access, with this effect being more pronounced among non-state-owned enterprises and firms in patent-intensive industries. Further analysis demonstrates that patent quality facilitates trade credit access by strengthening bargaining power and elevating corporate reputation. This research not only clarifies the mechanism that ultimately reinforces the operationalization of innovation-driven development frameworks by enhancing firms’ technological competitiveness and market credibility, but also enriches the channels through which patents influence corporate financing, and provides policy recommendations to advance patent quality development. These findings enable firms to leverage patent assets in reducing transaction costs and financing burdens.

## 1 Introduction

In recent years, Chinese firms have continuously enhanced their innovation capabilities, becoming a primary driver of patent output. By June 2025, Chinese firms held a total of 5.01 million invention patents. Among these, firms firmly occupy the dominant position in invention innovation: 524,000 firms held valid invention patents, totaling 3.727 million, which accounts for 74.4% of all valid domestic invention patents. However, the innovative substance of patents has not improved proportionally with the increase in quantity [1].Numerous studies have examined patent quality, defining and measuring it based on factors such as the patent application process, patent type, and scope of protection. While methods are not uniform, there is broad consensus that patent quality is diversified. High-quality patents possess greater technological and economic value, whereas low-quality patents may even stifle enterprise innovation.

Moreover, patent quality is a key factor determining a firm’s economic benefits and affects its stakeholders. Stakeholders assess a company’s future economic gains based on patent quality to decide how to collaborate with it. Suppliers and customers along the supply chain—the stakeholders most directly connected to the company—share in the economic benefits derived from high-quality patents. To establish partnerships with companies holding high-quality patents, customers and suppliers offer favorable terms, such as trade credit arrangements. These arrangements follow a “Buy Now, Pay Later” (BNPL) logic, thereby forming the company’s trade credit.

Trade credit serves as a vital source of liquidity for firms. It is an interest-free, unsecured form of supply chain financing and a key tool for coordinating production capacity and mitigating risk impacts among supply chain partners. For instance, upstream suppliers accept longer payment terms to ensure stable raw material supply, while downstream customers prepay to secure priority access to production capacity. During risk events, core firms can inject liquidity across the chain by extending payment terms. Obtaining trade credit financing essentially represents acquiring low-cost working capital. The greater the amount of trade credit financing secured, the more it enhances a firm’s operational flexibility.Therefore, investigating whether patent quality promotes access to trade credit explores the following scenario: whether firms consider patents when making collaboration decisions, and how this consideration influences their specific partnership strategies.

Based on this, this study examines whether and how patent quality facilitates access to trade credit financing, starting from the measurement of firm’s patent quality. The results show that patent quality significantly enhances corporate ability to obtain trade credit financing. This effect is particularly evident in non-state-owned firms with stronger innovation incentives and patent-intensive firms that highly value patents.The underlying reasons are that patent quality improves a firm’s supply chain concentration and corporate reputation, strengthening its bargaining power relative to suppliers. This, in turn, helps firms secure more favorable terms during transaction negotiations.

The potential contributions of this paper are twofold: (1) It extends the understanding of the economic consequences of improving corporate patent quality, supplementing existing literature. (2) It reveals what kind of firms—those possessing innovative outcomes with specific characteristics—gain more support from their supply chains. By measuring patent quality from the perspective of knowledge breadth, the findings demonstrate that patent quality serves as a reliable signal of a firm’s future market returns. This signal conveys positive information to upstream and downstream partners, giving firms an advantage in negotiations and enabling them to secure more favorable terms.

The remainder of this study proceeds as follows. Section 2 summarizes extant literature and develops testable hypotheses. Section 3 outlines the research design and methodology. Section 4 reports empirical findings. Section 5 provides concluding remarks and practical implications.

## 2 Literature review and research hypotheses

### 2.1 Literature review

Is patent quantity equivalent to patent quality? This question sparked scholarly interest in patent quality research. Studies show that patent quantity does not reflect actual patent value, and an increase in patents does not necessarily boost corporate profits [2].Patents fall into three categories: (1) invention patent, granted for new technical solutions related to products, processes, or their improvements; (2) utility model patent, granted for new technical solutions related to the shape, structure, or combination of products; (3) design patent, granted for new designs related to the shape, pattern, or combination of a product’s appearance.Technological sophistication decreases across these three categories.

During technological catch-up, latecomer countries often lower patent application barriers to encourage “minor” innovations like utility model patent. This aims to stimulate innovation enthusiasm and foster progress [3–6].However, utility model patent often involve imitating or modifying existing technologies from other countries. Their learning effects diminish over time. This passive innovation path, with its inherent technological limitations, leads to rising patent numbers without a proportional increase in quality. Significant differences exist in the innovative quality of different patents [7]. Therefore, the substance within a patent is key to determining its value. As outputs of innovation, it is the creation of high-quality patents—along with their innovative spillover effects and industrial applicability—that enables patents to effectively drive economic growth [8].

Existing research commonly measures patent quality in the following ways: First, from the perspective of legal validity. Patents are granted for a limited lifespan, protecting the holder’s rights only during their legal term. After expiration, this protection ceases. Therefore, patents are categorized as valid or invalid.prior studies consider invalid patents low-quality and use patent lifespan as a proxy for patent quality [9,10]. Second, from the perspective of market value impact. K Higham et al. [7] measure a patent’s financial value using stock market reactions shortly after its grant. Similarly, Long Xiaoning et al. [11] link patent value to stock market fluctuations.Thrid, from the perspective of citation impact. Patent citations indicate the continuity and cumulativeness of knowledge transfer. Frequent citation suggests greater potential societal benefit. Therefore, the number of citations a patent receives is also a key measure of its quality [12–17].

Additionally, some studies construct combined measures to assess patent quality. Such combined measures are necessary because patent quality is a multidimensional concept, encompassing factors like technical scope, economic value, and subsequent impact. A single indicator cannot capture this complexity [18]. For instance, forward citations reflect technological influence but not protection breadth or legal stability, while patent renewal indicates economic value but not technological breakthrough. Existing indicators used to comprehensively measure patent quality include: relative patent activity (RPA), patents per employee (PA/EMP), revealed international patent activity (REPA), grant rate (GP-Rate), valid patent rate (VP-Rate), citation rate (CIT), and technology scope (TS) [2,19]. The findings revealed that not all these metrics correlated with corporate economic benefits. These studies highlight the need to expand approaches to measuring patent quality, as different perspectives may yield different outcomes.

Existing literature proposes various definitions of patent quality. While methods differ, two underlying consensuses emerge: (1) The concept of patent quality is linked to the economic benefits a patent generates. Patents capable of creating economic value are considered high-quality; (2) There is no one-size-fits-all approach. Different measurement methods should be chosen based on the perspective of different stakeholders [7]. Based on this, this paper adopts the knowledge breadth method, applies it to Chinese data [20–26]. A firm’s patent knowledge breadth primarily refers to the complexity of the knowledge contained within a patent. Patents are carriers of innovative knowledge. The complexity of this knowledge is a key indicator of its technological sophistication. Furthermore, the more complex the knowledge within a patent, the harder it is to imitate and the lower its substitutability. This inevitably affects the monopoly power a firm gains over its innovative products through the patent protection system, thereby significantly impacting the economic benefits obtained by firms.

This method was chosen for two key reasons. First, existing studies have examined the similar concept of “Patent Scope” which measures the breadth of a patent’s coverage by the diversity and number of International Patent Classification (IPC) subclasses it spans within a specific technical field. Researches found patent scope significantly correlates with firm value, enhancing market competitiveness and value creation capacity [2,27]. This provides evidence that knowledge breadth aligns conceptually with patent quality. Second, suppliers and customers providing trade credit value a firm’s competitiveness and value creation capacity when choosing partners, as such firms offer sufficient economic returns. Therefore, using knowledge breadth as a measure of patent quality from the supply chain perspective is well-justified.

Research on the economic consequences of patent quality primarily focuses on firm value. Scholars have reached mixed conclusions on whether patent quality enhances firm value. Some results found a positive effect [28,29], while others obtained both positive and negative results using patent citations and patent concentration, respectively. [30] Chinese scholars have examined the impact of different patent types on firm value, with most studies concluding that invention patents contribute most significantly to value enhancement [31,32]. The positive effect of patent quality on firm performance has also been verified [33,34]. Research from an import-export perspective shows that improved patent quality promotes the export of high-quality products [23]. Furthermore, enhancing patent quality at the national level attracts foreign investment [35] and contributes to high-quality economic development [36].

Existing research on the economic consequences of patent quality primarily focuses on the impact within the firm itself. Studies examining the perspective of external stakeholders, such as supply chain partners, are less common and warrant further exploration. Therefore, this paper adopts the perspective of suppliers and customers —— stakeholders closely linked to the firm —— to investigate whether concessions granted by supply chain partners,which in the form of trade credit, vary based on the firm’s patent quality. Using sample data from Chinese firms, we empirically analyze this relationship. Our findings confirm that higher patent quality positively influences the amount of trade credit extended by suppliers and customers. These results hold significant both theoretical and practical importance for advancing innovation theory and improving management practices.

### 2.2 Research hypotheses

The Resource-Based View posits that a firm’s core competitiveness stems from its heterogeneous resources, encompassing both tangible and intangible assets. Knowledge, as a key intangible asset, forms the basis for differentiating a firm’s products or services [37]. Formally, patents represent the legally protected form of a firm’s knowledge assets. They possess characteristics such as limited duration, enforceability, transferability, and divisibility [38]. Patents effectively prevent competitors from imitating core technologies, thereby sustaining the firm’s differentiation advantage in product or service innovation [39,40]. Essentially, patents are an intermediate outcome of a firm utilizing its resources to create products or services. They serve as an objective measure of a firm’s technological capability and constitute an intangible asset [41]. Therefore, patent quality objectively reflects a firm’s technological strength. High-quality patents typically contain richer knowledge content and more complex technological combinations. This makes them harder to substitute, forming competitive barriers that act as a “moat” for sustainable competitive advantage. Consequently, this paper measures patent quality using the knowledge breadth method, following Zhang Jie and Zheng Wenping [22], to capture the technological diversity and depth embodied in patents.

From the perspective of trade credit decision logic, suppliers face two primary risks when extending credit: assessment bias due to information asymmetry, and losses arising from customer default [42]. Firms with high patent quality can mitigate these risks through a signaling effect, thereby strengthening the supplier’s willingness to provide credit. On the one hand, unlike intermediate indicators like R&D investment, patents are legally recognized outcomes of innovation. Their quality directly reflects a firm’s ability to convert knowledge into productive capabilities [43,44]. Information embedded in high-quality patents —— such as technical details and claim scope —— provides suppliers with reliable evidence to assess the firm’s technological strength and market potential, reducing decision uncertainty caused by opaque information. On the other hand, high patent quality signifies stronger technological barriers and market competitiveness, leading to greater profitability and cash flow stability for the firm’s products or services [45]. Additionally, the transferability and collateral value of patents offer suppliers additional repayment security, further mitigating concerns about default losses [46].

From the bargaining perspective, firms with high patent quality occupy a stronger position within the supply chain. This occurs for two key reasons. On the one hand, corporate technological barriers reduce alternative options for downstream customers, fostering stable downstream relationships. This makes upstream suppliers more willing to offer favorable credit terms to maintain the partnership, such as extending the payment period. On the other hand, product differentiation enabled by high-quality patents strengthens the firm’s bargaining power over downstream customers. To secure access to these less substitutable goods or services, customers are more likely to accept credit terms favorable to the supplier [47,48]. This dual bargaining advantage ultimately translates into increased trade credit financing.

In summary, high patent quality promotes greater access to trade credit by signaling firm competitiveness and lower default risk and strengthening the firm’s bargaining position within the supply chain. Based on this theoretical framework, we propose the first hypothesis:

*Hypothesis H1: The higher the quality of the patent, the more trade credit a firm can receive.*

As a core reflection of a firm’s technological strength and innovation capability, patent quality exerts a significant positive influence on the firm’s bargaining power within the supply chain. This, in turn, facilitates access to greater trade credit financing. Products and patents are key factors shaping supply chain relationships [49,50]. On one hand, high-quality patents support the uniqueness of products or services, reducing market substitution risks and giving the firm an advantageous position when negotiating with suppliers [28]; on the other hand, high patent quality signifies more prominent novelty, inventiveness, and practical applicability of technology, which can bring differentiated competitive advantages to the firm, enhance customer satisfaction and loyalty [51], reduce customer churn risk, and consequently grant greater say in setting transaction terms [52].

Additionally, the sustained profitability and risk resilience enabled by high patent quality further strengthen a firm’s bargaining confidence. Compared to low-quality patents, high-quality patents more readily translate into market value. The stable revenue generated from them provides assurance for repaying trade credit obligations, making counterparties more willing to extend credit support [53]. Furthermore, high-quality patents also serve to deter competitors, helping to consolidate the firm’s competitiveness. Consequently, by enhancing the firm’s bargaining power within the supply chain, patent quality ultimately promotes access to greater trade credit financing. Consequently, we frame the second hypothesis:

*Hypothesis H2: Higher patent quality strengthens corporate bargaining power, facilitating enhanced access to trade credit.*

Patent quality serves as a crucial signal to the market of a firm’s innovation capability and contractual reliability, significantly enhancing its corporate reputation. A strong reputation is a key prerequisite for obtaining trade credit financing. Signaling theory indicates that in markets with information asymmetry, firms need to convey credible information about their quality to counterparties through persuasive signals [54]. High patent quality helps build a firm’s positive image and market standing, which is vital for its long-term development. On one hand, high-quality patents signal the firm’s entrepreneurial spirit and robust creative capacity [55,56]. They also imply the firm’s commitment to intellectual property protection and long-term orientation [27]. This signal is interpreted by stakeholders like suppliers and customers as indicative of the firm’s operational stability and growth potential, thereby accumulating positive market reputation. For instance, firms holding core high-quality patents are more likely to be perceived as “reliable partners” with lower perceived default risk [14].

On the other hand, a strong credit reputation reduces debt costs [57]. To protect their positive market reputation, firms tend to place greater emphasis on credit management. The patent system grants inventors temporary monopoly rights, enabling them to disclose detailed information publicly [58]. This disclosure enhances corporate reputation [59]. The patent system also formalizes innovation as an intangible asset. Higher patent quality translates into greater intangible asset value and higher collateral value [60], providing strong repayment assurance for trade credit [61] and thereby strengthening credit reputation. At its core, trade credit is a trust-based short-term financing arrangement [42]. Firms with better reputations enjoy greater trust in their ability to fulfill obligations, making counterparties more willing to offer favorable credit terms. Suppliers may proactively extend payment periods or increase credit limits to maintain relationships with high-reputation firms. Alternatively, firms can leverage their reputation to negotiate more advantageous credit terms. Furthermore, during short-term liquidity shocks, reputable firms find it easier to obtain credit extensions from counterparties, indirectly enhancing the sustainability of their trade credit financing [62]. Based on this theoretical framework, we propose the third hypothesis:

*Hypothesis H3: Higher patent quality improves the reputation of the firm, facilitating enhanced access to trade credit.*

## 3 Research design

### 3.1 Sample and data

Our study selects Chinese A-share listed companies from 2006 to 2023 as a sample for research. The patent information of listed companies comes from the State Intellectual Property Office of China, the data of trade credit comes from the CSMAR database, and the data of other control variables are from the CSMAR database.

### 3.2 Variable definitions

#### 3.2.1 Independent Variable.

The independent variable in the study is patent quality (*Patentquality*), which includes both invention and utility patents. Referring to the approach based on knowledge breadth by Zhang and Zheng [22] and Li et al. [23], we use patent quality indicator by measuring the number of different types of patents held by a firm. Specifically, Chinese patents are classified using the IPC (International Patent Classification) format, which categorizes patents according to technical subjects for ease of retrieval and management. The patent classification number format is “section - main class - subclass - main group - subgroup,” where “section” is represented by uppercase letters A-H, totaling 8 sections; “main class” is indicated by two Arabic numerals, with each main class representing a technical field and being a part of a section; “subclass” is denoted by uppercase letters A to Z, and sometimes double letters are used to avoid confusion, with subclasses being part of a main class. To enhance the accuracy of patent quality estimation, the study defines the firm’s patent knowledge breadth at the main group level, following the calculation method of the Herfindahl Index, and calculates patent quality in the following manner:


$$\begin{array}{c} {Patentquality}_{it}=\text{1}-\sum {\left(\frac{Z_{imt}}{Z_{it}}\right)}^{\text{2}} \end{array}$$

Among them, Z_imt_ is the cumulative number of invention and utility patent applications filed by enterprise i under group m as of year t, and Z_it_ is the cumulative number of patent applications filed by firm i under all groups as of year t. The larger the value of *Patentquality*, the greater the knowledge breadth of the firm’s patent, and the higher the patent quality.

Patents are categorized into three types: Invention Patents, Utility Model Patents, and Design Patents. Following the approach of Li Hong et al. [23], this study calculates this indicator only for Invention Patents and Utility Model Patents. Design Patents are excluded for two reasons: (1) their numbering system is entirely different from the other two types, making them incompatible with the same calculation method; and (2) they generally contain a relatively lower level of technological innovation, and thus cannot fully reflect the knowledge complexity embodied in patents.

In practice, firms often file applications for both invention patents and utility model patents simultaneously. Due to their differing levels of complexity, utility model patents grant protection faster, typically within 6–12 months, allowing firms to secure patent protection earlier and support enforcement actions. Invention patents undergo stricter and lengthier examination, usually taking 2–4 years. When filing identical content for both types, if the invention patent passes examination, the firm may declare abandonment of the already granted utility model patent right to obtain the invention patent grant, achieving “seamless transition”. If the invention patent ultimately fails to pass, the firm still retains the utility model patent as a safeguard, ensuring it does not end up empty-handed. Should the subsequent invention patent’s content and scope of protection differ from the utility model, abandonment becomes unnecessary, enabling “product + method” dual protection.

In other words, from the perspective of safeguarding corporate interests, invention patents and utility model patents can form a combined strategy to jointly create a “moat” effect. The key to achieving this effect lies in obtaining patent grants. Patent application refers to the process where an applicant submits technical documentation to the China National Intellectual Property Administration (CNIPA), requesting examination and grant of patent rights. At this stage, the application merely expresses the firm’s intent to secure rights; legal protection has not yet been conferred, preventing the prohibition of others’ implementation or the initiation of infringement lawsuits. However, the patent application does grant the right, after 18 months of publication, to claim reasonable compensation for the use of the technology by others. Patent grant refers to the process where, after substantive examination confirms the application meets authorization criteria, CNIPA issues a patent certificate conferring exclusive rights upon the applicant. At this stage, the enterprise enjoys full patent rights, including exclusive implementation, licensing, assignment, and litigation enforcement. Thus, the protective effect of the patent form on corporate interests varies across different stages of patent protection. The fundamental purpose for suppliers and clients to assess an firm’s patent quality remains evaluating the extent to which patents can generate benefits for the firm. Whether the submitted patent applications ultimately receive approval may be key to answering this question.

Therefore, this study distinguishes between nodes in the patent application process, separately calculating the quality of enterprise patents in the application phase and the granted phase. It examines whether differences exist in the trade credit financing obtained by firms when the protective efficacy of patents varies.

#### 3.2.2 Dependent variable.

The dependent variable of this paper is trade credit (*TC*). The study refers to the research of Lu Zhengfei and Yang Deming [63], Giannetti M et al. [64] and Fang Mingyue [65] to define trade credit access (TC) = (accounts payable + notes payable + advance receipts)/ total assets.

#### 3.2.3 Control variable.

Drawing on established empirical models from relevant literature, this study controls for various factors that may influence trade credit financing acquisition. The controlled variables include: firm size, operating cash flow, firm leverage, growth potential, bank financing, asset structure, profitability, firm maturity, governance structure, executive compensation, separation of powers, and industry competition. Table 1 presents the comprehensive variable selection and their respective measurement methodologies.

**Table 1:** Variable measurement.

Type	Name	Symbol	Measurement
**Dependent variable**	Patent quality	*Patentquality*	Based on the patent quality calculated based on the knowledge width, Patentquality1 indicates that the firm applies for patent (Patent Application), and Patentquality2 indicates that the firm has obtained patent (Granted Patent)
**Independent variable**	Trade credit	TC	(Accounts Payable + Notes Payable + Advance Receipts)/ Total Assets
**Moderating variable**	Supply chain concentration	SCC	(Proportion of purchases from the top 5 suppliers + proportion of sales to the top 5 customers)/2
	Corporate reputation	Rep	Company’s reputation score calculated by factor analysis
**Control variable**	Firm size	*Size*	Ln (Total Assets)
	Operating cash flow	*OCF*	Net cash flow from operating activities/total assets
	Firm leverage	*Lev*	total liabilities/total assets
	Growth potential	*Growth*	Growth rate of operating income
	Bank financing	*Bank*	Total bank borrowings/liabilities
	Asset structure	*Mortgage*	(Fixed Assets + Inventory)/ Total Assets
	Firm maturity	*Age*	Ln (years of age + 1)
	Governance structure	*Executive*	Ln (Total number of directors)
	Executive compensation	*Comp*	Ln (total compensation of the top three senior executives)
	Separation of powers	*Separation*	Separation of powers
	Industry competition	*HHI*	The sum of the squares of the ratio of the operating income of each company in the industry to the total operating income of the industry
	Profitability	*ROA*	Net profit after tax/total assets

### 3.3 Model construction

Our study examines the impact of patent quality on corporate trade credit access through the following empirical model:


$$TC_{it}=\beta_{\text{0}}+\beta_{\text{1}}\;Patentquality_{it}+Controls_{it}+ind_{i}+year_{t}+\varepsilon_{it}$$ (1)

Among them, i represents the enterprise, t represents the year, the independent variable is *Patentquality*, and the dependent variable is trade credit (*TC*). Controls represents control variables. In order to avoid the potential effects of industry differences and temporal trends, the fixed effects of industry (*ind*) and year (*year*) are controlled in the model.

### 3.4 Descriptive statistics

In order to exclude the effect of extreme values, all continuous variables are winsorized at the quantile levels of 1 and 99. Table 2 summarizes the data characteristics. The average quality scores for patent application (*Patentquality1*) and granted patent (*Patentquality2*) were 0.786 and 0.746, with variations (standard deviations) of 0.274 and 0.311, indicating that the sample companies showed a similar trend in the breadth of knowledge covered by these two types of patents. On average, the level of trade credit in the sample interval accounted for 16.5% of the total assets.

**Table 2:** Descriptive statistics.

	N	Mean	SD	Max	p50	Min
*Patentquality1*	24338	0.786	0.274	0.991	0.897	0
*Patentquality2*	24338	0.746	0.311	0.992	0.880	0
*TC*	24338	0.165	0.114	0.548	0.139	0.00600
*Size*	24338	22.38	1.389	26.38	22.17	19.36
*Separation*	24338	5.113	7.668	28.52	0	0
*OCF*	24338	0.0450	0.0660	0.248	0.0440	−0.179
*Mortgage*	24338	0.381	0.168	0.818	0.375	0.0250
*HHI*	24338	0.183	0.161	1	0.130	0.0370
*Growth*	24338	0.180	0.417	3.038	0.116	−0.612
*Executive*	24338	2.291	0.243	2.890	2.303	1.609
*Comp*	24338	14.35	0.820	16.39	14.36	11.93
*Bank*	24338	0.338	0.211	0.822	0.341	0
*Age*	24338	3.226	0.209	3.664	3.258	2.639
*Lev*	24338	0.475	0.197	1.016	0.479	0.0580
*ROA*	24338	0.0360	0.0630	0.223	0.0350	−0.255

## 4 Results and discussion

### 4.1 Baseline regression analysis

Table 3 reports the baseline regression results. Columns (1) to (2) examine the impact of patents in the application stage on trade credit financing, while columns (3) to (4) investigate the effect of granted patents. The regression results show that the coefficients of *Patentquality1* and *Patentquality2* in columns (1) to (4) are all significantly positive at the 1% level. This indicates that both patent applications and granted patents positively influence enterprises access to trade credit financing, supporting Hypothesis H1 of this study. Specifically, a 1% increase in the quality of patent applications raises the ratio of trade credit to total assets by 0.0211 percentage points, while a 1% improvement in the quality of granted patents increases this ratio by 0.0192 percentage points. This suggests that when transacting with firms, suppliers and clients consider the actual quality of their patents, showing greater preference for partners with complex knowledge structures and lower imitability. Such enterprises demonstrate more stable market competitiveness, making them desirable partners across the supply chain.

**Table 3:** Baseline regression result.

	Patent Application		Granted Patent	
	(1)	(2)	(3)	(4)
	TC	TC	TC	TC
*Patentquality1*	0.0359***	0.0211***		
	(0.0025)	(0.0019)		
*Patentquality2*			0.0345***	0.0192***
			(0.0023)	(0.0017)
*Size*		−0.0042***		−0.0043***
		(0.0005)		(0.0005)
*Separation*		0.0003***		0.0003***
		(0.0001)		(0.0001)
*OCF*		−0.0429***		−0.0423***
		(0.0081)		(0.0081)
*Mortgage*		0.0228***		0.0228***
		(0.0034)		(0.0034)
*HHI*		0.0158***		0.0162***
		(0.0057)		(0.0057)
*Growth*		0.0025*		0.0026**
		(0.0013)		(0.0013)
*Executive*		−0.0008		−0.0007
		(0.0020)		(0.0020)
*Comp*		0.0007		0.0008
		(0.0008)		(0.0008)
*Bank*		−0.2464***		−0.2465***
		(0.0026)		(0.0026)
*Age*		−0.0126***		−0.0125***
		(0.0024)		(0.0024)
*Lev*		0.3873***		0.3873***
		(0.0033)		(0.0033)
*ROA*		0.1253***		0.1276***
		(0.0097)		(0.0097)
*Constant*	0.1368***	0.1570***	0.1393***	0.1601***
	(0.0021)	(0.0139)	(0.0018)	(0.0140)
Industry	Yes			
Year	Yes			
N	24338	24338	24338	24338
Adjusted R^2^	0.2672	0.6136	0.2678	0.6136

*Note: Standard errors in parentheses.*

*p < 0.1.

**p < 0.05.

***p < 0.01.

*The subsequent forms are the same.*

When patents are in the application stage, they represent a firm’s innovation potential and technological R&D capability, yet lack full legal protection and market recognition. For suppliers and clients, pending patent applications serve as signals of future development—particularly when involving innovative technologies with market-leading potential. They may perceive the firm’s innovation capacity and potential as guarantees for future growth. The act of filing patent applications demonstrates the firm’s confidence in its innovations, providing compelling justification. Consequently, firms in the patent application phase can secure more favorable credit terms from suppliers or clients, explaining the positive relationship observed for *Patentquality1*.

Once a patent is granted, it establishes definitive legal protection and gains broad market recognition. For suppliers and clients, granted patents serve not only as direct proof of a firm’s innovative capability but also as symbols of its market competitiveness and technological stability. Granted patents confer explicit legal rights, strengthening intellectual property protection and thereby reducing transaction risks for partners. Thus, firms holding granted patents typically receive greater support in trade credit financing. Suppliers and clients may view such firms as possessing significant technological barriers to entry, along with stronger repayment capacity and lower default risk.

The baseline regression results reveal that the coefficient of *Patentquality2* is slightly lower than that of *Patentquality1*. This may reflect suppliers’ and clients’ relatively cautious stance toward granted patents. Although granted patents offer enhanced legal safeguards and market recognition, an firm’s actual market performance may be influenced by factors such as technology commercialization capability and market demand. The distinction between Patentquality1 and Patentquality2 reflects differential risk assessments by suppliers and clients based on patent status: Applications signal future potential and may influence short-term credit decisions, while granted patents provide stronger legal guarantees and market value, typically delivering more stable and robust credit support. Overall, while differences exist between the two stages, they are not statistically significant; both the legal status and market recognition of patents play critical roles in trade credit financing.

### 4.2 Heterogeneity tests

#### 4.2.1 Heterogeneity analysis of patent intensity of the industry.

In this study, following the *Catalogue of Patent-Intensive Industries (2016)* issued by the State Intellectual Property Office of China and building upon the methodology of Shan Xiaoguang et al. [66], industries classified under codes C26, C27, C34, C35, C36, C37, C38, C39, C40, and I65 are identified as patent-intensive industries, while the remaining industries are categorized as non-patent-intensive. Table 4 shows how results differ across industries with varying levels of patent intensity.

**Table 4:** Heterogeneity analysis based on industry patent intensity.

	Patent Application		Granted Patent	
	Intensive	Non-Intensive	Intensive	Non-Intensive
	(1)	(2)	(3)	(4)
	TC	TC	TC	TC
*Patentquality1*	0.0252***	0.0180***		
	(0.0028)	(0.0025)		
*Patentquality2*			0.0239***	0.0149***
			(0.0025)	(0.0023)
*Size*	−0.0022***	−0.0057***	−0.0023***	−0.0057***
	(0.0007)	(0.0007)	(0.0007)	(0.0007)
*Separation*	0.0002**	0.0003***	0.0002**	0.0003***
	(0.0001)	(0.0001)	(0.0001)	(0.0001)
*OCF*	−0.0691***	−0.0111	−0.0689***	−0.0100
	(0.0110)	(0.0116)	(0.0110)	(0.0116)
*Mortgage*	0.0424***	0.0078	0.0423***	0.0080*
	(0.0048)	(0.0048)	(0.0048)	(0.0048)
*HHI*	0.0393***	0.0008	0.0377***	0.0017
	(0.0111)	(0.0067)	(0.0111)	(0.0067)
*Growth*	−0.0009	0.0066***	−0.0007	0.0066***
	(0.0017)	(0.0020)	(0.0017)	(0.0020)
*Executive*	0.0006	−0.0048	0.0008	−0.0046
	(0.0026)	(0.0030)	(0.0026)	(0.0030)
*Comp*	0.0010	−0.0013	0.0010	−0.0013
	(0.0011)	(0.0012)	(0.0011)	(0.0012)
*Bank*	−0.2660***	−0.2219***	−0.2660***	−0.2220***
	(0.0034)	(0.0039)	(0.0034)	(0.0039)
*Age*	−0.0166***	−0.0168***	−0.0162***	−0.0169***
	(0.0031)	(0.0036)	(0.0031)	(0.0036)
*Lev*	0.4352***	0.3164***	0.4347***	0.3167***
	(0.0043)	(0.0050)	(0.0043)	(0.0050)
*ROA*	0.1395***	0.1056***	0.1420***	0.1076***
	(0.0124)	(0.0150)	(0.0124)	(0.0150)
*Constant*	0.1057***	0.2657***	0.1084***	0.2684***
	(0.0184)	(0.0210)	(0.0184)	(0.0211)
Industry	Yes			
Year	Yes			
N	13188	11150	13188	11150
Adjusted R^2^	0.6335	0.6060	0.6337	0.6057
p-value	0.010***		0.000***	

Columns (1) ~ (2) and (3) ~ (4) in Table 4 present the regression results for firms applying for patents and obtaining authorized patents in patent-intensive industries and non-patent-intensive industries, respectively. The patent quality coefficients in columns (1) ~ (4) are all positive and significant at the 1% level, indicating that both patent application and granted patent can promote the acquisition of trade credit in both patent-intensive and non-patent-intensive industries. This matches our earlier findings. Further Fisher combination test results show that both groups can reject the null hypothesis at the 1% level, indicating that the impact of patent quality on trade credit differs between patent-intensive and other industries. Specifically, companies in patent-intensive industries see bigger changes in trade credit when their patents cover broader knowledge areas.

Given the significantly divergent results above, this paper argues that in patent-intensive industries, patents are a key manifestation of innovation output capability [67]. Patent quality represents the qualitative dimension of technological innovation, directly linked to the industry’s core competitiveness, technology transfer efficiency, and sustainable development capacity. It is one of the key characteristics distinguishing these industries from others. Consequently, patents confer a greater competitive advantage within patent-intensive industries. This result further supports the close connection between patent quality and a firm’s market competitiveness.

#### 4.2.2 Heterogeneity analysis of nature of business ownership.

Studies show private companies invest more in innovation and create patents more efficiently than state-owned firms [68]. To explore how company ownership affects these patterns, we analyzed different business types separately. The detailed findings are in Table 5.

**Table 5:** Heterogeneity analysis based on enterprise ownership.

	Patent Application		Granted Patent	
	SOEs	Non-SOEs	SOEs	Non-SOEs
	(1)	(2)	(3)	(4)
	TC	TC	TC	TC
*Patentquality1*	0.0105***	0.0217***		
	(0.0030)	(0.0026)		
*Patentquality2*			0.0103***	0.0202***
			(0.0027)	(0.0024)
*Size*	−0.0093***	−0.0057***	−0.0094***	−0.0058***
	(0.0008)	(0.0008)	(0.0008)	(0.0008)
*Separation*	0.0006***	0.0001	0.0006***	0.0001
	(0.0001)	(0.0001)	(0.0001)	(0.0001)
*OCF*	−0.0560***	−0.0371***	−0.0559***	−0.0370***
	(0.0136)	(0.0105)	(0.0136)	(0.0105)
*Mortgage*	0.0004	0.0405***	0.0003	0.0400***
	(0.0056)	(0.0046)	(0.0056)	(0.0046)
*HHI*	0.0149	0.0118	0.0154	0.0123
	(0.0108)	(0.0076)	(0.0107)	(0.0076)
*Growth*	0.0033	0.0034**	0.0035	0.0033*
	(0.0023)	(0.0017)	(0.0023)	(0.0017)
*Executive*	0.0056*	−0.0052**	0.0055	−0.0053**
	(0.0034)	(0.0026)	(0.0034)	(0.0026)
*Comp*	−0.0042***	−0.0008	−0.0042***	−0.0009
	(0.0014)	(0.0010)	(0.0014)	(0.0011)
*Bank*	−0.3024***	−0.2297***	−0.3025***	−0.2303***
	(0.0045)	(0.0033)	(0.0045)	(0.0033)
*Age*	−0.0215***	−0.0134***	−0.0216***	−0.0131***
	(0.0046)	(0.0029)	(0.0046)	(0.0029)
*Lev*	0.4419***	0.3921***	0.4421***	0.3924***
	(0.0058)	(0.0046)	(0.0058)	(0.0046)
*ROA*	0.2096***	0.0725***	0.2102***	0.0764***
	(0.0187)	(0.0122)	(0.0187)	(0.0122)
*Constant*	0.3680***	0.2161***	0.3711***	0.2204***
	(0.0258)	(0.0189)	(0.0258)	(0.0189)
Industry	Yes			
Year	Yes			
N	9223	11978	9223	11978
Adjusted R^2^	0.6824	0.5955	0.6825	0.5956
p-value	0.010***		0.000***	

Columns (1) ~ (2) and (3) ~ (4) in Table 5 respectively present the regression results of the impact of patent application and granted patent on trade credit acquisition in state-owned firms (SOEs) and non-state-owned firms (non-SOEs). The patent quality coefficients in columns (1) ~ (4) are all positive and significant at the 1% level, which is consistent with the benchmark test results of this paper. This indicates that the conclusion that higher patent quality leads to greater trade credit financing applies to both SOEs and non-SOEs. Specifically, there are differences in the size of the coefficients between columns (1) ~ (2) and columns (3) ~ (4). The coefficients for SOEs are smaller than those for non-SOEs. Further Fisher combination test results show that both groups can reject the null hypothesis at the 1% level, indicating that the impact of patent quality on trade credit differs between SOEs and non-SOEs. This effect is more pronounced in non-SOEs, which have stronger innovation and patenting tendencies.

The significantly divergent results above are attributed to the fact that state-owned enterprises (SOEs) benefit from government support. Their motivation to pursue profits through technological innovation is weaker compared to self-sustaining non-SOEs. For SOEs, patent quality plays a more complementary rather than decisive role. Their core competitiveness stems primarily from government backing, whereas non-SOEs’ core competitiveness derives more from technology-driven value creation.

When comparing different types of patents within the same category of firms, the difference between *Patentquality1* in column (1) and *Patentquality2* in column (3) is not substantial, while the gap between *Patentquality1* in column (2) and *Patentquality2* in column (4) is statistically insignificant. This indicates a minimal distinction between patents application and granted patents, with the observed difference being notably smaller than in the previous test. Consequently, supply chain partners focus primarily on whether patent applications exist when engaging with state-owned versus non-state-owned firms. However, when comparing patent-intensive industries to non-patent-intensive industries, their evaluation extends to a deeper analytical level, demonstrating greater sensitivity to the legal status of patents.

### 4.3 Mechanism tests

This study investigates the signaling function of patent quality in trade credit acquisition through the dual lenses of corporate bargaining power and reputation. A firm’s bargaining power directly correlates with its ability to establish trust within supply chain networks, fostering stronger trading partnerships with upstream and downstream entities while reducing dependency on specific customers or suppliers. To operationalize this concept, the research employs supply chain concentration (SCC) as a proxy for bargaining power, adopting the measurement methodology established by Patatoukas [69] and subsequently refined by Fang Hongxing et al. [70] and Zhao Ziqiang et al. [71]. Specifically, SCC is calculated as the average of two components: the proportion of purchases from the top five suppliers and the proportion of sales to the top five customers. An increase in this value indicates higher supply chain concentration.

Corporate Reputation (Rep) is calculated using the method of Guan Kaolei and Zhang Rui [72]. This method involves selecting the industry rankings of corporate assets, revenue, net profit, and value from the perspectives of consumers and society. It also includes the debt-to-asset ratio, current ratio, and long-term debt ratio from the perspective of creditors; earnings per share and dividend per share from the perspective of shareholders; and whether the company is audited by the Big Four accounting firms. Additionally, from the perspective of the enterprise, the sustainable growth ratio and the ratio of independent directors are considered. These 12 reputational evaluation indicators are used to calculate the corporate reputation score in factor analysis, which is then ranked from low to high on a scale of 1–10.

Table 6 shows the results of the mechanism tests. Columns (1) ~ (2) indicate a negative correlation between invention patent quality and supply chain concentration at the 1% significance level. Specifically, higher patent quality is associated with lower supply chain concentration, thereby enhancing the bargaining power of firms. This result supports Hypothesis 2 of the study. Meanwhile, the results in columns (3) ~ (4) reveal a positive correlation between utility model patent quality and corporate reputation at the 1% significance level. That is, higher patent quality is linked to better corporate reputation, which in turn supports Hypothesis 3 of the study. Therefore, improving patent quality can facilitate access to trade credit by strengthening bargaining power and enhancing corporate reputation.

**Table 6:** Mechanism tests.

	Bargaining Power		Reputation	
	(1)	(2)	(3)	(4)
	SCC	SCC	Rep	Rep
*Patentquality1*	−4.9554***		0.1422***	
	(0.4377)		(0.0344)	
*Patentquality2*		−4.7169***		0.1458***
		(0.3986)		(0.0315)
Size	−3.2600***	−3.2315***	1.8423***	1.8406***
	(0.1119)	(0.1121)	(0.0094)	(0.0094)
Separation	0.0075	0.0075	0.0090***	0.0090***
	(0.0132)	(0.0132)	(0.0011)	(0.0011)
OCF	−7.9740***	−8.0700***	1.4541***	1.4582***
	(1.7814)	(1.7807)	(0.1475)	(0.1474)
Mortgage	−0.2153	−0.2142	−0.1899***	−0.1897***
	(0.7447)	(0.7445)	(0.0623)	(0.0623)
HHI	2.1749	2.0420	−0.8176***	−0.8150***
	(1.3697)	(1.3693)	(0.1031)	(0.1030)
Growth	3.2921***	3.2402***	0.0856***	0.0866***
	(0.2799)	(0.2798)	(0.0239)	(0.0239)
Executive	0.3940	0.3965	−0.3972***	−0.3967***
	(0.4203)	(0.4202)	(0.0361)	(0.0361)
Comp	−0.8932***	−0.8827***	0.2838***	0.2837***
	(0.1781)	(0.1781)	(0.0149)	(0.0149)
Bank	7.5384***	7.5544***	−0.5871***	−0.5875***
	(0.5684)	(0.5682)	(0.0469)	(0.0469)
Age	−2.4444***	−2.5186***	0.1501***	0.1514***
	(0.4897)	(0.4897)	(0.0430)	(0.0430)
Lev	−4.3089***	−4.3154***	0.1977***	0.1991***
	(0.7225)	(0.7222)	(0.0602)	(0.0602)
ROA	−4.3852**	−4.7744**	12.8477***	12.8646***
	(2.0642)	(2.0627)	(0.1778)	(0.1777)
Constant	128.0210***	127.1592***	−39.2634***	−39.2292***
	(3.0089)	(3.0120)	(0.2545)	(0.2549)
Industry	Yes			
Year	Yes			
N	18798	18798	23933	23933
Adjusted R^2^	0.2753	0.2757	0.7924	0.7925

Compared to existing research, these findings further supplement the micro-level mechanisms of how patents influence the supply chain. Prior studies predominantly focus on the impact of patent quantity and citation counts on corporate financing, yielding inconsistent conclusions. this paper demonstrates a significant marginal effect of patent quality. This also explains why some firms with large patent portfolios struggle to obtain financing support: low-quality patents fail to confer a substantial bargaining advantage. Regarding the reputation mechanism, while existed research [72] explain the positive effect of CEO reputation on trade credit financing, this study extends reputation to the firm level, providing further evidence of reputation’s influence on trade credit. Moreover, it verifies that patent quality is a significant antecedent of firm reputation.

### 4.4 Robustness tests

#### 4.4.1 Replace patent quality measurements.

We use the number of patent citations as a measure of patent quality to test the robustness. The number of patent citations (*Times*) is calculated as the cumulative number of patent citations in the current year and previous years. Table 7 presents the results of the regression analysis, substituting the number of patent citations for the breadth of patent knowledge. The coefficients of Times in columns (1) ~ (2) are significantly positive at the 1% level, indicating that the more times a patent is cited, the more trade credit access the firm can obtain. This finding supports the hypothesis of the study. Detailed control variable coefficients are provided in S1 Table of the Supporting Information.

**Table 7:** Robustness tests1.

	(1)	(2)
	TC	TC
Times	0.0040***	0.0009***
	(0.0002)	(0.0002)
Controls	Yes	
Industry	Yes	
Year	Yes	
N	20537	20537
Adjusted R^2^	0.2814	0.6084

#### 4.4.2 Replace trade credit measurements.

We replace the calculation method of trade credit for robustness testing, defining the calculation method of trade credit as the ratio of accounts payable, notes payable, advances from customers to net profit (*TC_Res)*. Table 8 shows the regression results. In columns (1) ~ (2), the coefficients of *Patentquality1* are significantly positive at the 1% and 5% levels, respectively. In columns (3) ~ (4), the coefficients of *Patentquality2* remain positive but differ in significance levels. This difference may be due to the utility model patent trap among Chinese enterprises. Specifically, utility model patents do not cover innovations in methods, processes, or materials, only protecting product structures rather than core technologies. During application, utility model patents undergo only formal examination and novelty checks, lacking in-depth evaluation of inventiveness, resulting in authorized patents with varying technological content. Additionally, the protection period for utility model patents is 10 years, far shorter than the 20 years for invention patents. Consequently, authorized patents contain relatively less knowledge content and have weaker effects on enhancing corporate bargaining power and reputation compared to invention patents, which may be reflected in the significance levels of the test coefficients. Overall, the results of this robustness test support the hypothesis of this paper. Detailed control variable coefficients are provided in S2 Table of the Supporting Information.

**Table 8:** Robustness tests2.

	Patent Application		Granted Patent	
	(1)	(2)	(3)	(4)
	TC_Res	TC_Res	TC_Res	TC_Res
*Patentquality1*	1.6054***	1.0707**		
	(0.4944)	(0.4927)		
*Patentquality2*			1.3638***	0.4815
			(0.4527)	(0.4521)
*Controls*	Yes			
*Industry*	Yes			
*Year*	Yes			
*N*	24,338	24,338	24,338	24,338
*Adjusted R* ^*2*^	0.0432	0.1058	0.0431	0.1057

### 4.5 Endogeneity tests

Enterprises with substantial innovation investments are more likely to obtain patents, suggesting potential sample selection bias in this study. To address this, we employ the Heckman two-stage selection model, incorporating the proportion of high and new-technology enterprises (*Htec*) certified in the firm’s industry as an instrumental variable. The results are presented in Table 9. Columns (1) and (3) report the first-stage selection equation results, while columns (2) and (4) show the second-stage outcome equation results. In the first stage, the coefficients of *Htec* are statistically significant at the 1% level, indicating that both the application and grant quality of enterprise patents are significantly influenced by peer firms’ technological levels. A higher concentration of high and new-technology enterprises in the industry creates competitive pressure, prompting firms to produce higher-quality patents. The significant inverse Mills ratio confirms non-ignorable sample selection bias. After including imr in the second-stage outcome equation, the regression results remain robustly significant at the 1% level, validating this study’s hypotheses.

**Table 9:** Heckman two-satge selection model.

	Patent Application		Granted Patent	
	Selection	Outcome	Selection	Outcome
	(1)	(2)	(3)	(4)
	Indicator	TC	Indicator	TC
*Patentquality1*		0.0220***		
		(10.7722)		
*Patentquality2*				0.0196***
				(11.0651)
Htec	0.9078***		0.7476***	
	(13.5735)		(12.2447)	
imr		0.0200***		0.0154***
		(5.2243)		(4.1583)
Controls	Yes			
Industry	Yes			
Year	Yes			
N	27519	21226	30906	23627
R^2^		0.614		0.611

## 5 Conclusion and recommendations

This study investigates the impact of corporate patent quality on trade credit access, utilizing data from Chinese A-share listed companies over the period from 2006 to 2023. The empirical finds that better patent quality helps firms get more trade credit. This benefit is stronger for non-state-owned enterprises and firms within patent-intensive industries. Further analysis reveals that patent quality works by boosting a company’s bargaining power and reputation. Specifically, firms with higher patent quality exhibit stronger bargaining power, as evidenced by lower supply chain concentration, and enjoy a better corporate reputation, which in turn facilitates access to trade credit. Based on the aforementioned conclusions, this study proposes the following implications:

(1) Strengthen Intellectual Property Protection and Enhance Patent Quality

Governments should intensify intellectual property protection efforts, refine patent examination systems, and elevate patent quality to prevent low-quality patent proliferation. Concurrently, firms should be encouraged to conduct high-quality R&D activities, file, and maintain superior patents, thereby reinforcing the reliability of patents as resource-based positive signals.

(2) Optimize Enterprise Innovation Management

Firms should improve the conversion efficiency of R&D investments into outputs, increase patent application and grant success rates. By enhancing patent quality, firms can not only bolster their technological innovation capabilities and safeguard innovation outcomes but also strengthen bargaining power with upstream and downstream partners.

(3) Prioritize Corporate Reputation Building:

Firms should emphasize reputation cultivation by transmitting positive market signals through high-quality patent activities, thereby elevating corporate image and market recognition. A robust corporate reputation facilitates stable cooperative relationships within supply chains, reduces transaction costs, and improves access to trade credit.

(4) Develop Differentiated Strategies Aligned with Enterprise Characteristics:

For non-state-owned enterprises and patent-intensive industries, greater emphasis should be placed on patent quality improvement, as these entities benefit more substantially from such enhancements. For state-owned enterprises, alongside patent quality elevation, further reforms to innovation incentive mechanisms are warranted to stimulate innovation vitality and enhance patent commercialization capabilities.

(5) Policy Support and Guidance

When formulating industrial policies, governments should account for firm heterogeneity by providing tailored support. For patent-intensive industries, tax incentives and fiscal subsidies could incentivize increased R&D investments and patent quality improvements. For non-state-owned enterprises, intellectual property services and strengthened protection measures would aid in advancing patent commercialization competencies.

This study expands the understanding of how intellectual property rights, particularly the quality of patents, affect a company’s access to finance. This study enriches the literature on the economic consequences of patent quality by demonstrating that higher patent quality enhances access to trade credit financing. At the same time, the study provides empirical evidence on the mechanisms by which patent quality affects trade credit —— by enhancing bargaining power and enhancing corporate reputation. This finding highlights the importance of intangible assets in shaping a firm’s financial decisions,supporting resource-based perspective. In addition, the study contributes to the trade credit literature by identifying patent quality as a key determinant of a firm’s ability to obtain trade credit. These results reveal how innovation and ownership of ideas boost trust in supply chain partnerships.

Nonetheless, this study has certain limitations: it defines patent quality solely from the “knowledge breadth” perspective. However, patent quality is a multidimensional concept, also encompassing technological innovativeness, legal stability, and commercial value. Relying on a single dimension may fail to fully capture the true quality of patents, potentially leading to an incomplete reflection of the relationship between patent quality and trade credit financing and overlooking unexplored mechanisms. Future research will attempt to introduce multidimensional indicators for a comprehensive assessment of patent quality, for example: introducing citation frequency and the number of claims from the technological dimension to reflect the scope of protection; adding litigation success rate and maintenance period from the legal dimension to reflect legal stability; combining licensing revenue and collateral loan amount from the commercial dimension to reflect commercial value. Through multidimensional measurement, the relationship between patent quality and trade credit financing can be revealed more accurately.

In conclusion, this study underscores the critical role of patent quality in shaping corporate access to trade credit financing. By enhancing bargaining power and corporate reputation, high-quality patents provide firms with a competitive edge in obtaining trade credit. These findings offer valuable insights for both policymakers and corporate decision-makers, highlighting the need to prioritize innovation and intellectual property protection to foster economic growth and corporate success. Future research could further explore the mechanisms and boundary conditions of this relationship, as well as its applicability across different contexts and firm types.

## Supporting information

S1 Table Robustness tests1. We use the number of patent citations as a measure of patent quality to test the robustness. The number of patent citations (Times) is calculated as the cumulative number of patent citations in the current year and previous years. This Table presents the results of the regression analysis, substituting the number of patent citations for the breadth of patent knowledge with detailed control variable coefficients.
(DOCX)

S2 Table Robustness tests2. We replace the calculation method of trade credit for robustness testing, defining the calculation method of trade credit as the ratio of accounts payable, notes payable, advances from customers to net profit (*TC_Res)*. This Table shows the regression results with detailed control variable coefficients.
(DOCX)

S3 Table Heckman two-satge selection model. We use the proportion of high and new-technology enterprises (Htec) certified in the firm’s industry as an instrumental variable. The results are presented in this Table.
(DOCX)

S1 Data Supporting Information of Original Data. We provide the original data for reference, including all variables required for the analyses in the paper.
(XLSX)
