# Supplementary material for: Patent quality and trade credit: Based on the perspective of knowledge breadth
Source: PLoS One. 2025 Oct 31;20(10):e0335515. doi: 10.1371/journal.pone.0335515 (PMC12578242; doi:10.1371/journal.pone.0335515)
Supplement: S1 Table — We use the number of patent citations as a measure of patent quality to test the robustness. The number of patent citations (Times) is calculated as the cumulative number of patent citations in the current year and previous years. This Table presents the results of the regression analysis, substituting the number of patent citations for the breadth of patent knowledge with detailed control variable coefficients. (DOCX) [file pone.0335515.s001.docx]

**S1 Table. Robustness tests1**

|  | (1) | (2) |
| --- | --- | --- |
|  | TC | TC |
| Times | 0.0040*** | 0.0009*** |
|  | (0.0002) | (0.0002) |
| Size |  | -0.0040*** |
|  |  | (0.0006) |
| Separation |  | 0.0003*** |
|  |  | (0.0001) |
| OCF |  | -0.0446*** |
|  |  | (0.0090) |
| Mortgage |  | 0.0245*** |
|  |  | (0.0037) |
| HHI |  | 0.0211*** |
|  |  | (0.0067) |
| Growth |  | 0.0025* |
|  |  | (0.0014) |
| Executive |  | -0.0016 |
|  |  | (0.0021) |
| Comp |  | 0.0000 |
|  |  | (0.0009) |
| Bank |  | -0.2448*** |
|  |  | (0.0028) |
| Age |  | -0.0108*** |
|  |  | (0.0025) |
| Lev |  | 0.3794*** |
|  |  | (0.0036) |
| ROA |  | 0.1401*** |
|  |  | (0.0105) |
| Constant | 0.1607*** | 0.1751*** |
|  | (0.0007) | (0.0161) |
| Industry Fixed Effect | Yes | Yes |
| Year Fixed Effect | Yes | Yes |
| N | 20537 | 20537 |
| Adjusted R^2^ | 0.2814 | 0.6084 |

*Note: Standard errors in parentheses.*

**p < 0.1,*

***p < 0.05,*

****p < 0.01.*
