# Supplementary material for: Patent quality and trade credit: Based on the perspective of knowledge breadth
Source: PLoS One. 2025 Oct 31;20(10):e0335515. doi: 10.1371/journal.pone.0335515 (PMC12578242; doi:10.1371/journal.pone.0335515)
Supplement: S2 Table — We replace the calculation method of trade credit for robustness testing, defining the calculation method of trade credit as the ratio of accounts payable, notes payable, advances from customers to net profit (TC_Res). This Table shows the regression results with detailed control variable coefficients. (DOCX) [file pone.0335515.s002.docx]

**S2 Table. Robustness tests2**

|  | **Patent Application** | | **Granted Patent** | |
| --- | --- | --- | --- | --- |
|  | (1) | (2) | (3) | (4) |
|  | TC_Res | TC_Res | TC_Res | TC_Res |
| *Patentquality1* | 1.6054*** | 1.0707** |  |  |
|  | (0.4944) | (0.4927) |  |  |
| *Patentquality2* |  |  | 1.3638*** | 0.4815 |
|  |  |  | (0.4527) | (0.4521) |
| *Size* |  | 0.9876*** |  | 1.0100*** |
|  |  | (0.1348) |  | (0.1353) |
| *Seperation* |  | -0.0292* |  | -0.0288* |
|  |  | (0.0158) |  | (0.0158) |
| *OCF* |  | -15.1328*** |  | -15.0920*** |
|  |  | (2.1187) |  | (2.1187) |
| *Mortgage* |  | 2.1561** |  | 2.1513** |
|  |  | (0.8952) |  | (0.8953) |
| *HHI* |  | -0.1306 |  | -0.1130 |
|  |  | (1.4891) |  | (1.4892) |
| *Growth* |  | -2.1435*** |  | -2.1458*** |
|  |  | (0.3429) |  | (0.3430) |
| *Executive* |  | 1.2076** |  | 1.2204** |
|  |  | (0.5202) |  | (0.5202) |
| *Comp* |  | -3.0919*** |  | -3.0810*** |
|  |  | (0.2145) |  | (0.2145) |
| *Bank* |  | -5.9921*** |  | -6.0078*** |
|  |  | (0.6736) |  | (0.6736) |
| *Age* |  | 1.5938** |  | 1.5865** |
|  |  | (0.6190) |  | (0.6192) |
| *Lev* |  | 25.0862*** |  | 25.0742*** |
|  |  | (0.8630) |  | (0.8631) |
| *ROA* |  | 12.5094*** |  | 12.6535*** |
|  |  | (2.5467) |  | (2.5458) |
| *Constant* | 7.0253*** | 11.8949*** | 7.2706*** | 11.7182*** |
|  | (0.4077) | (3.6650) | (0.3592) | (3.6706) |
| *Industry* | Yes | | | |
| *Year* | Yes | | | |
| *N* | 24,338 | 24,338 | 24,338 | 24,338 |
| *Adjusted R^2^* | 0.0432 | 0.1058 | 0.0431 | 0.1057 |

*Note: Standard errors in parentheses.*

**p < 0.1,*

***p < 0.05,*

****p < 0.01.*
