# Supplementary material for: Patent quality and trade credit: Based on the perspective of knowledge breadth
Source: PLoS One. 2025 Oct 31;20(10):e0335515. doi: 10.1371/journal.pone.0335515 (PMC12578242; doi:10.1371/journal.pone.0335515)
Supplement: S3 Table — We use the proportion of high and new-technology enterprises (Htec) certified in the firm’s industry as an instrumental variable. The results are presented in this Table. (DOCX) [file pone.0335515.s003.docx]

**S3 Table. Heckman two-satge selection model**

|  | **Patent Application** | | **Granted Patent** | |
| --- | --- | --- | --- | --- |
|  | Selection | Outcome | Selection | Outcome |
|  | (1) | (2) | (3) | (4) |
|  | Indicator | TC | Indicator | TC |
| *Patentquality1* |  | 0.0225*** |  |  |
|  |  | (11.6321) |  |  |
| *Patentquality2* |  |  |  | 0.0196*** |
|  |  |  |  | (11.0651) |
| Htec | 0.7476*** |  | 0.7476*** |  |
|  | (12.2447) |  | (12.2447) |  |
| imr |  | 0.0157*** |  | 0.0154*** |
|  |  | (4.2330) |  | (4.1583) |
| Size | 0.3279*** | -0.0025*** | 0.3279*** | -0.0026*** |
|  | (27.8355) | (-4.3000) | (27.8355) | (-4.4559) |
| Separation | -0.0017 | 0.0003*** | -0.0017 | 0.0003*** |
|  | (-1.3255) | (4.3811) | (-1.3255) | (4.3887) |
| OCF | -0.0689 | -0.0417*** | -0.0689 | -0.0411*** |
|  | (-0.4389) | (-5.1491) | (-0.4389) | (-5.0762) |
| Mortgage | -0.1029 | 0.0215*** | -0.1029 | 0.0215*** |
|  | (-1.5148) | (6.2036) | (-1.5148) | (6.2008) |
| HHI | -0.1915 | 0.0162*** | -0.1915 | 0.0165*** |
|  | (-1.5421) | (2.8217) | (-1.5421) | (2.8785) |
| Growth | -0.0788*** | 0.0020* | -0.0788*** | 0.0021* |
|  | (-3.5272) | (1.6985) | (-3.5272) | (1.7442) |
| Executive | 0.0689 | 0.0003 | 0.0689 | 0.0004 |
|  | (1.5243) | (0.1534) | (1.5243) | (0.1969) |
| Comp | 0.1204*** | 0.0012 | 0.1204*** | 0.0013 |
|  | (6.8718) | (1.4935) | (6.8718) | (1.5441) |
| Bank | 0.2460*** | -0.2452*** | 0.2460*** | -0.2453*** |
|  | (4.6477) | (-92.9307) | (4.6477) | (-92.9476) |
| Age | -0.6949*** | -0.0155*** | -0.6949*** | -0.0153*** |
|  | (-11.2247) | (-6.2361) | (-11.2247) | (-6.1723) |
| Lev | -0.5132*** | 0.3777*** | -0.5132*** | 0.3777*** |
|  | (-7.8566) | (111.5836) | (-7.8566) | (111.5657) |
| ROA | 0.7596*** | 0.1308*** | 0.7596*** | 0.1339*** |
|  | (3.8633) | (13.6620) | (3.8633) | (13.9859) |
| Constant | -7.0348*** | 0.1199*** | -7.0348*** | 0.1239*** |
|  | (-16.8671) | (7.5038) | (-16.8671) | (7.7447) |
| Industry Fixed Effect | Yes | Yes | Yes | Yes |
| Year Fixed Effect | Yes | Yes | Yes | Yes |
| N | 30906 | 23627 | 30906 | 23627 |
| R^2^ |  | 0.612 |  | 0.611 |

*Note: Standard errors in parentheses.*

**p < 0.1,*

***p < 0.05,*

****p < 0.01.*
